# Parliament and the Profession

**Published:** 1919-12-13

**Authors:** 


### Parliament and the Profession.

### Tuberculous Ex-officers.

THE Minister of Pensions stated that a scheme for providing outdoor employment for ex-officers is under consideration.

### Pensions: Medical Staff.

GENERAL CROFT was informed by Sir J. Craig, speaking for the Minister of Pensions, that the rate of pay for medical men on Examining Boards has been increased to £1 11s. 6d. per session, and that of full-time officers remains at £1 1s. It is considered inequitable that the difference between occasional employment, which cannot be counted upon from day to day, and regular and full employment, which can be counted upon, should be marked by a difference in the rate of pay. All fees of assessors, however, are being reviewed in the light of enhanced cost of living.

### Ministry of Health: Women Employees.

DR. ADDISON stated that fifteen women are employed by the Ministry of Health in an administrative capacity. Three are permanent employees with salaries from £150 to £260, plus a war bonus, and twelve temporary workers with salaries from £170 to £250.

